# Medial opening-wedge high tibial osteotomy with microfracture in treatment of varus medial compartmental knee osteoarthritis: clinical outcomes and second-look arthroscopic results

**DOI:** 10.3389/fbioe.2023.1247165

**Published:** 2023-09-21

**Authors:** Yunpeng Bai, Binhui Lin, Miao Wang, Haoliang Ding, Weibing Sun, Jian Sun

**Affiliations:** Department of Orthopaedic Surgery, Jiading Branch of Shanghai General Hospital, Shanghai Jiao Tong University School of Medicine, Shanghai, China

**Keywords:** high tibial osteotomy, arthroscopy, medial compartmental osteoarthritis, cartilage regeneration, microfracture

## Abstract

**Objective:** This study aimed to investigate the clinical outcomes of medial opening high tibial osteotomy (MOWHTO) combined with arthroscopic microfracture in the treatment of varus medial compartmental knee osteoarthritis and to assess cartilage regeneration using second-look arthroscopy.

**Methods:** This study involved 86 patients (86 knees) who underwent MOWHTO and microfracture from August 2016 to August 2020, including 15 men and 71 women with an average age of 55.3 ± 7.6 years (range, 42–71 years). The patients underwent a second-look arthroscopy to evaluate the status of cartilage regeneration at the time of plate removal, an average of 2 years after the initial osteotomy. Clinical and radiological examinations were performed preoperatively and at the final follow-up visit. The radiologic evaluation included the weight-bearing line ratio (WBL ratio), mechanical femorotibial angle (FTA), medial proximal tibial angle (MPTA), posterior tibial slope angle (PTS) and Kellgren-Lawrence (KL) grade. Clinical outcomes were assessed using the Knee Society score (KSS) and International Knee Documentation Committee (IKDC) scores. Arthroscopic findings were assessed by macroscopic evaluation of cartilage repair according to the International Cartilage Repair Society (ICRS) grading system.

**Results:** The mean KSS and IKDC scores significantly improved at the final follow-up compared to the scores obtained preoperatively (*p* < 0.05). At the time of plate removal, a second-look arthroscopic examination showed that the ICRS grade of the medial femoral condyle was as follows: grade I −11 cases, grade II -56, grade III-12, and grade IV-7, and cartilage regeneration was seen in 85% of knees (73/86). The ICRS grade of medial tibial plateau was grade I-12 cases, grade II-44, grade III-22, and grade IV-8, and cartilage regeneration was seen in 63% of knees (54/86). Significant differences were observed between cartilage regeneration and clinical outcomes (*p* < 0.05). Clinical results were better in the good cartilage regeneration group (grades I and II) than were in the poor cartilage regeneration group (grades III and IV).

**Conclusion:** MOWHTO combined with arthroscopic microfracture can effectively improve clinical outcomes in the treatment of varus medial compartmental knee osteoarthritis. Cartilage regeneration can be promoted by correcting varus deformities, which affect clinical outcomes.

## 1 Introduction

High tibial osteotomy (HTO) is an established surgical method for the treatment of medial compartmental knee osteoarthritis combined with varus deformity (Akizuki et al., 2008; Amendola and Bonasia, 2010; Jin et al., 2020). Over the last 2 decades, with the development of internal fixation implants and surgical techniques, medial opening wedge high tibial osteotomy (MOWHTO) has been widely used and has achieved good clinical results (Sta et al., 2003; Kyung et al., 2015; MacLeod et al., 2018). By correcting varus deformities, the weight-bearing line can be restored, the knee joint load can be transferred from the medial to the lateral compartment, the load on the medial knee compartment can be reduced, the mechanical environment of the medial compartment of the knee can be improved, and cartilage regeneration can be achieved for self-remodeling (Sterett et al., 2010; Parker et al., 2011; Jung et al., 2014). However, the grading of cartilage regeneration status using secondary microscopy and its relationship with clinical outcomes remains unclear.

Arthroscopic microfractures are often used to treat cartilage damage, stimulating the bone marrow through multiple drilling holes in the subchondral bone. The bone marrow stem cells and cytokines exuded from the micropores have a certain role in promoting the regeneration of degenerative cartilage (Solheim et al., 2016; Neri et al., 2020; Chung et al., 2021). However, whether MOWHTO combined with arthroscopic microfractures can improve clinical outcomes and effectively promote degenerative cartilage regeneration and the relationship between cartilage regeneration and knee function improvement remains unclear.

In this study, we retrospectively analyzed 86 cases of MOWHTO combined with arthroscopic microfracture followed by second-look arthroscopy to evaluate clinical outcomes and arthroscopic results and to determine whether cartilage regeneration status was associated with postoperative outcomes.

## 2 Materials and methods

### 2.1 Patients

A total of 86 cases with varus knee arthritis treated with MOWHTO combined with knee arthroscopic microfractures between August 2016 and August 2020 were enrolled in this study. All patients were diagnosed with varus medial compartmental arthritis and were ineffective by conservative treatment for more than 3 months. All surgeries were performed by an experienced surgeon, using the same internal fixation implants and surgical techniques. All patients underwent internal implant removal surgery 2 years after the MOWHTO and second-look arthroscopy simultaneously.

The inclusion criteria were as follows: 1) age ≥40 years, 2) medial compartmental knee osteoarthritis with varus deformity, varus alignment >3°, 3) knee range of flexion ≥120°, 4) knee flexion contracture <15°, and 5) a stable knee without anterior cruciate ligament deficiency.

The exclusion criteria were as follows: 1) knee infection, traumatic arthritis, and rheumatoid arthritis; 2) lateral compartment osteoarthritis; 3) anatomic varus alignment ≥10°, 4) moderate to severe patellofemoral arthritis; 5) cartilage procedures other than microfractures; 6) follow-up period of <2 years; 7) incomplete clinical or radiological data. This study was approved by the institutional review board of our hospital, and all patients provided informed consent before surgery.

### 2.2 Surgical procedure and postoperative rehabilitation

A standing full-length lower limb anteroposterior radiograph was obtained to measure the angle of the varus and determine the degree of correction preoperatively. We planned to shift the lower extremity mechanical axis to pass through the Fujisawa point (62.5% of the width of the tibial condyle from the medial edge to the lateral edge) (Fujisawa et al., 1979; Matsushita et al., 2021). Before the MOWHTO, arthroscopic examinations were routinely performed to determine the cartilage status of the medial, lateral, and patellofemoral compartments of the knee joints. Articular cartilage status is graded during arthroscopy according to the International Cartilage Repair Society (ICRS) scoring system. Joint debridement was routinely performed and at the discretion of the surgeon, meniscectomy or repair was performed for existing meniscal tears. During the microfracture procedure, loose cartilage flaps were first cleaned using a planer and radiofrequency to create a stable and clear cartilage edge. Holes were made using a 2.0-mm Kirschner wire at the exposed subchondral bone with a depth of 3–5 mm and 2–3 mm apart. Of these 86 patients, 38 underwent meniscectomy, 20 underwent meniscal repair, and all underwent microfracture.

After arthroscopy, a biplanar MOWHTO was performed according to the AO International Knee Expert Group (Lobenhoffer and Agneskirchner, 2003). Under fluoroscopic guidance, the proximal end of the alignment rod was located at the center of the hip joint and the distal end was located at the center of the ankle joint. The surgeon then confirmed that the rod passed through a point located 62.5° lateral to the medial cortex of the tibial plateau. The osteotomy site was fixed with a locking plate (APS), and the allograft bone was transplanted when the osteotomy gap width was >10 mm. The drainage tube was maintained for 2–3 d until the drainage volume reached <50 ml.

All the patients were rehabilitated under the guidance of a professional rehabilitation therapist. Passive knee motion exercises and muscle-strengthening exercises were initiated on the first day after surgery. After removing the drainage tube, partial weight-bearing ambulation was performed using a walker. Six weeks after surgery, full weight-bearing was allowed with a cane, and at 12 weeks after surgery, activities such as running, jumping, and climbing were allowed.

Second-look arthroscopy was performed approximately 2 years after surgery. When radiography confirmed that the osteotomy site achieved bone union, internal fixation removal surgery was performed along with second-look arthroscopy, during which knee cartilage status was evaluated and cartilage regeneration was graded according to the ICRS scoring system.

### 2.3 Radiological evaluation

At the preoperative and final follow-up, standardized anteroposterior and lateral views and full-length lower limb radiographs were obtained in a standing position. The radiologic evaluation included the WBL ratio passing through a point lateral from the medial edge of the tibial plateau (WBL ratio), mechanical femorotibial angle (FTA), medial proximal tibial angle (MPTA), posterior tibial slope angle (PTS), and Kellgren-Lawrence grade (KL). We calculated the correction angle of the lower limb weight-bearing line after surgery compared with that preoperatively.

### 2.4 Clinical evaluation

The Knee Society Score (KSS) and International Knee Documentation Committee (IKDC) scores were used to evaluate the patients’ knee function preoperatively and at the final follow-up. Two senior surgeons independently performed the clinical evaluations.

### 2.5 Cartilage assessment

The medial femoral condyle and tibial plateau articular cartilage were evaluated by arthroscopy before and approximately 2 years after MOWHTO, and the ICRS grading system was used to score three aspects: defect repair degree, marginal fusion degree, and microscopic general performance. Based on the total score, grades I, II, III, and IV were normal, close to normal, abnormal, and severely abnormal, respectively. The medial femoral condyle and the medial tibial platform were used for cartilage regeneration. Grades I and II were defined as good cartilage regeneration and grades III and IV were defined as poor cartilage regeneration.

### 2.6 Statistical analysis

SPSS21.0 statistical software (IBM, Armonk, NY, United States) was used for statistical analysis. The measurement data were all in line with the normal distribution by the normality test, and the data were expressed as mean ± standard deviation; the paired t-test was used for comparison before and after surgery. The rank sum test was used before and after surgery, and bilateral α = 0.05 was considered the test level.

## 3 Results

**FIGURE 1:**
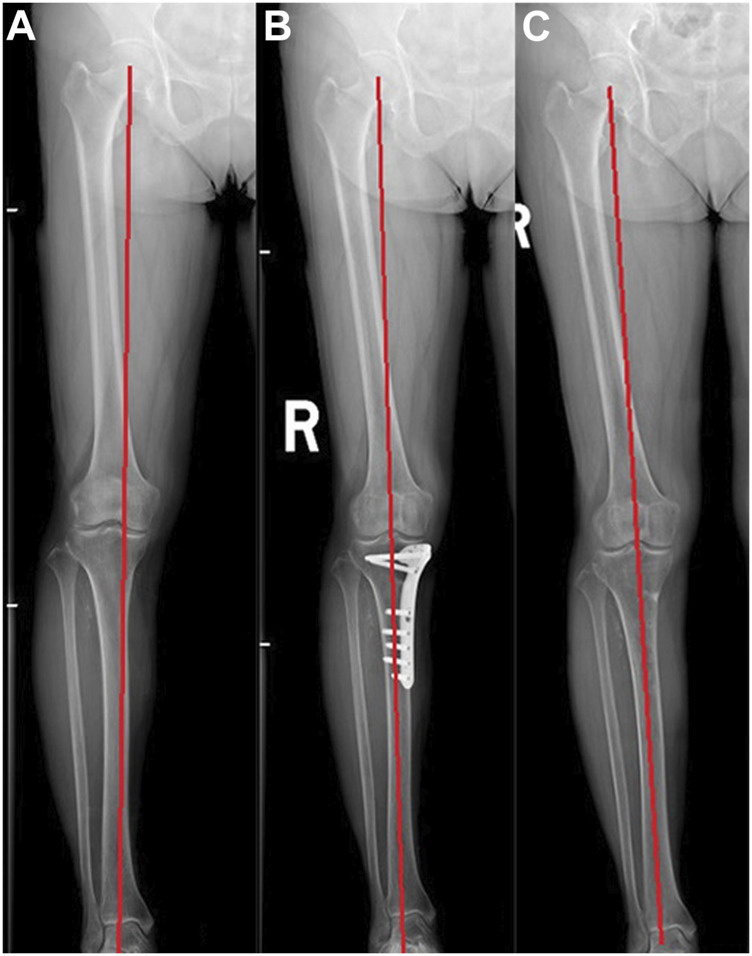
A 60-year-old woman underwent medial opening wedge high tibial osteotomy (MOWHTO) followed by second-look arthroscopy at implant removal. **(A)** Preoperative whole standing lower limb image showing varus limb alignment. **(B)** The varus deformity of lower limb was corrected after MOWHTO. **(C)** Postoperative whole standing lower limb image showing well-maintained limb alignment after implant removal.

The average follow-up time of the 86 patients was 3.9 years (range, 2–6 years). All patients underwent secondary arthroscopy, and the average interval between the primary and secondary surgery was 22.4 months (22–26 months). At the last follow-up, the KSS and IKDC scores were significantly higher than those before surgery (*p* < 0.05); the WBL ratio, FTA, and MPTA were significantly improved compared with preoperative values (*p* < 0.05); there was no significant difference between PTS before and after MOWHTO (*p* > 0.05); there was no significant difference between KL grade before and after MOWHTO (*p* > 0.05) (Table 1; Figure 1). All patients achieved bone union without complications such as incision infection, bone nonunion, failure of internal fixation, or rupture of internal fixation.

**TABLE 1:** Radiological and clinical outcomes before and after open wedge high tibial osteotomy combined with microfracture.

Variable	preoperative	Latest follow-up	*p*-value
WBL ratio (%)	27.9 ± 5.7	61.5 ± 6.4	<0.001
FTA	182.4 ± 1.7	176.6 ± 1.2	0.011
MPTA	84.6 ± 2.5	90.2 ± 1.8	0.024
PTS	7.0 ± 3.4	7.5 ± 2.3	0.453
KL grade			0.645
2	46	50	
3	40	36	
KSS	65.3 ± 4.8	90.4 ± 5.4	<0.001
KSSf	67.5 ± 7.4	91.6 ± 8.2	<0.001
IKDC	55.6 ± 3.2	83.1 ± 6.1	<0.001

WBL, weight bearing line; FTA, lateral femorotibial angle; MPTA, medial proximal tibial angle; PTS, posterior tibial slope angle; KL, Kellgren-Lawrence grade; KSS, knee society knee score; KSSf, Knee Society function score; IKDC, international knee documentation committee scores.

**FIGURE 2:**
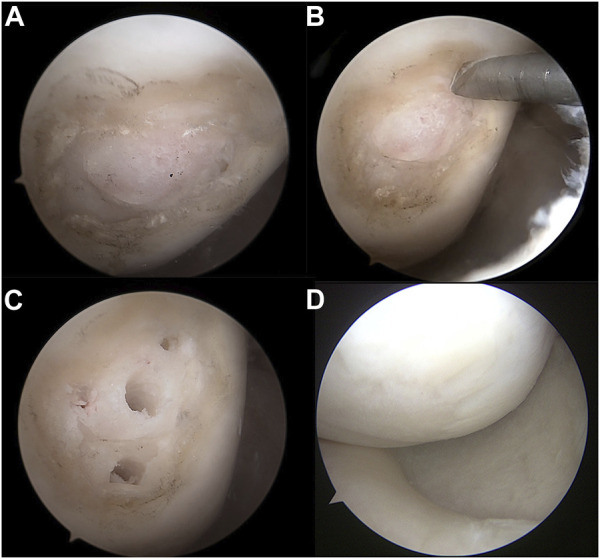
**(A)** The articular surface showed eburnation during MOWHTO. **(B, C)** Kirschner wire was used to perform mircrofracture during MOWHTO. **(D)** The articular surface showed normal coverage with fibrocartilage during second-look arthroscopy 2 years after MOWHTO.

Arthroscopic results showed that during the second operation, the ICRS grade of medial femoral condyle cartilage was grade I- 11 cases, grade II-56 cases, grade III- 12 cases, and grade IV- 7 cases, of which 73 cases (85%) showed cartilage regeneration. The ICRS grade of medial tibial plateau cartilage was grade I- 12 cases, grade II- 44 cases, grade III- 22 cases, and grade IV- 8 cases, of which cartilage regeneration was visible in 54 cases (63%) (Table 2; Figure 2). The clinical outcomes of patients with good cartilage regeneration status (grades I and II) were significantly better than for those with a poor cartilage regeneration status (grades III and IV) (*p* < 0.05) (Table 3).

**TABLE 2:** Arthroscopic outcomes according to ICRS grade.

	Initial	second look	Improved	Unimproved	*p*-value
MFC (%)			73 (85)	13 (15)	<0.001
Ⅰ	0	11			
Ⅱ	21	56			
Ⅲ	42	12			
Ⅳ	23	7			
MTP (%)			54 (63)	32 (37)	<0.001
Ⅰ	3	12			
Ⅱ	24	44			
Ⅲ	35	22			
Ⅳ	24	8			

ICRS, international cartilage repair society grading system; MFC, medial femoral condyle; MTP, medial tibial plateau.

**TABLE 3:** Clinical outcomes of open wedge high tibial osteotomy combined with microfracture according to articular cartilage status.

Variable	Good status (Ⅰ,Ⅱ)	Poor status (Ⅲ,Ⅳ)	*p*-value
total, n	56	30	
male, n (%)	9 (16.1)	6 (20.0)	0.767
mean age, yrs	54.5 ± 5.6	56.4 ± 6.2	0.352
mean BMI	25.1 ± 1.8	24.8 ± 2.7	0.265
with comorbidities, n (%)	35 (62.5)	18 (60.0)	0.820
KL grade			0.823
2	32	18	
3	24	12	
KSS	92.3 ± 2.8	85.6 ± 3.2	0.001
KSSf	92.8 ± 4.4	86.1 ± 2.7	0.015
IKDC	86.7 ± 1.3	78.4 ± 5.2	0.023

BMI, body mass index; KL, Kellgren-Lawrence grade; KSS, knee society knee score; KSSf, Knee Society function score; IKDC, international knee documentation committee scores.

## 4 Discussion

In this study, knee function in all patients after MOWHTO was significantly improved by secondary arthroscopy, and cartilage regeneration was observed in most patients in this group. Cartilage regeneration is associated with improved knee function and patients with better cartilage regeneration have thus improved knee function.

The main symptoms of varus knee arthritis are medial knee pain and limited mobility. The main causes are excessive weight bearing in the medial compartment due to varus deformity, secondary cartilage degeneration, meniscal tears, and osteoarthritis. According to existing research results, HTO surgery corrects knee varus deformity, adjusts the lower limb weight-bearing line to a mild valgus position (2°–6° valgus) (Lee et al., 2014; Yang et al., 2021), and reduces the weight bearing of the medial compartment, thereby achieving pain relief and functional improvement. Whether HTO can improve the cartilage status of the medial compartment of the knee has been the focus of research in recent years. Some studies have reported some degree of cartilage regeneration in the medial knee compartment after HTO using secondary microscopy, and various studies have reported varying rates of cartilage regeneration. Kim et al. (Kim et al., 2017) included 104 cases of HTO without cartilage treatment, and a second microscopic examination found that according to the ICRS grade, 51.9% of the medial femoral condyle and 34.6% of the medial tibial plateau had cartilage regeneration. A study of 62 knees by Kim et al. (Kim et al., 2017) found cartilage regeneration in 29% of knee joints using secondary microscopy. In a study by Kumagai et al. (Kumagai et al., 2017) that included 131 knee HTOs procedures, cartilage regeneration was found in 71% of the medial femoral condyles and 51% of the medial tibial plateaus. However, the factors affecting cartilage regeneration remain controversial. Kim et al. (Kim et al., 2017) found that medial femoral condylar cartilage regeneration was associated with a smaller preoperative mFTA and lower BMI, medial tibial platform cartilage regeneration was associated with younger age and larger correction angles, and patients with low BMI were more likely to achieve medial compartment cartilage regeneration through multivariate regression analysis. Kim et al. (Kim et al., 2017) found that severe arthrosis, the existence of a bipolar lesion, and marked postoperative joint line obliquity were adverse factors affecting cartilage regeneration. Kumagai et al. found that a low BMI, medial femoral condyle, higher ICRS grade, and overcorrection before surgery were favorable factors affecting cartilage regeneration.

However, whether arthroscopic microfracture effectively promotes cartilage regeneration remains controversial. Recent studies have shown that the association between HTO and microfractures is not associated with cartilage regeneration. Jung et al. (Jung et al., 2015) included 61 cases of HTO divided into two groups: one group of 30 cases of HTO + microfracture and the other group of 31 cases of simple HTO, through clinical scoring and secondary microscopic examination to evaluate joint cartilage regeneration; the results showed that there was no significant difference in clinical function or cartilage regeneration grade between the two groups. A study by Lee et al. (Lee et al., 2019) included 87 cases of HTO divided into two groups: a group of 57 patients who underwent HTO + microfractures and a group of 30 patients who underwent simple HTO, and after 2 years of follow-up, MRI evaluation found that cartilage regeneration in the microfracture group was better than that in the HTO alone group, but there was no significant difference in clinical and imaging results between the two groups. However, several studies have shown that micro-fractures promote cartilage damage (Eldracher et al., 2014; Iida et al., 2021; Stachel et al., 2022). It is generally believed that drilling in the cartilage defect area can stimulate the bone marrow, and the bone marrow stem cells exuded from the micropores can differentiate into chondroblasts under the influence of certain physical and chemical factors and then form new cartilage. However, the specific molecular biological mechanism remains unclear. At present, some studies have used biologics to promote cartilage repair, such as platelet-rich plasma, collagen scaffolds, mesenchymal stem cells, etc. (Kim et al., 2017; Chung et al., 2021; Kim et al., 2021; Lee et al., 2021; Yang et al., 2022), but there are some complications such as infection and rejection, and the certainty of safety and efficacy needs to be further evaluated. Microfracture technology has the advantages of simple operation, low cost, and no special complications. Microfracture technology was used in 86 cases, and secondary microscopic examination revealed a high cartilage regeneration rate.

Most studies have found that the cartilage regenerated after HTO surgery is the fibrocartilage, and there is a gap in its biomechanical and biochemical properties compared to the original hyaline cartilage. Therefore, whether fibrocartilage regeneration after HTO surgery is related to improvement in clinical function has been a hot topic in recent research. Several clinical studies have shown that cartilage regeneration after HTO is not correlated with clinical outcomes (Jung et al., 2015; Kim et al., 2017; Kim et al., 2017). Recent studies demonstrated a correlation between cartilage regeneration after HTO and clinical outcomes. A study by Yanget al. (Yang et al., 2022), which included 155 HTO patients, divided the ICRS grades of secondary cartilage regeneration into good (I, II) and poor (III, IV), and found that the clinical function of the group with good cartilage regeneration status was better than that of the poor group, which is consistent with the results of this study.

This study had some limitations. First, it was a retrospective study with small sample size and no control group. Second, the second microscopic examination was performed 2 years after HTO; however, whether there was a further change in the cartilage status after long-term follow-up is unclear. Third, the ICRS scoring system, an evaluation method for assessing the cartilage regeneration status, is based on general observations, and more accurate tissue biopsy-based methods are required to assess the cartilage regeneration status. Fourth, the intrinsic mechanism of cartilage regeneration and related factors have not been studied, and further research is needed to determine the correlation between cartilage regeneration and knee biomechanics and between cartilage regeneration and the knee microenvironment.

## 5 Conclusion

MOWHTO combined with microfracture could improve clinical and radiologic outcomes in the treatment of medial compartmental osteoarthritis with varus deformity. Cartilage regeneration of the medial femoral condyle and medial tibial plateau can be expected during second-look arthroscopy. The regenerative cartilage status was correlated with clinical outcomes after MOWHTO; however, the intrinsic mechanism should be explored in future research.

## Data Availability

The original contributions presented in the study are included in the article/supplementary material, further inquiries can be directed to the corresponding authors.
